# Image-Guided Ablations in Patients with Recurrent Renal Cell Carcinoma

**DOI:** 10.3390/jcm12154902

**Published:** 2023-07-26

**Authors:** Gaetano Aurilio, Giovanni Mauri, Duccio Rossi, Paolo Della Vigna, Guido Bonomo, Gianluca Maria Varano, Daniele Maiettini, Maria Cossu Rocca, Elena Verri, Daniela Cullurà, Franco Nolé, Franco Orsi

**Affiliations:** 1 Division of Cancer Prevention and Genetics, Istituto Europeo di Oncologia IEO, European Institute of Oncology IRCCS, 20122 Milan, Italy; gaetano.aurilio@ieo.it; 2 Division of Interventional Radiology, Istituto Europeo di Oncologia IEO, European Institute of Oncology IRCCS, 20122 Milan, Italy; giovanni.mauri@ieo.it (G.M.); paolo.dellavigna@ieo.it (P.D.V.); guido.bonomo@ieo.it (G.B.); gianluca.varano@ieo.it (G.M.V.); daniele.maiettini@ieo.it (D.M.); franco.orsi@ieo.it (F.O.); 3 Department of Oncology, Istituto Europeo di Oncologia IEO, European Institute of Oncology IRCCS, 20122 Milan, Italy; maria.cossurocca@ieo.it (M.C.R.); elena.verri@ieo.it (E.V.); daniela.cullura@ieo.it (D.C.); franco.nole@ieo.it (F.N.)

**Keywords:** renal cell carcinoma, ablation, metastasis

## Abstract

Renal cell carcinoma (RCC) is one of the most frequently diagnosed tumors and a leading cause of death. The high risk of local recurrence and distant metastases represent a significant clinical issue. Different image-guided ablation techniques can be applied for their treatment as an alternative to surgery, radiotherapy or systemic treatments. A retrospective analysis was conducted at our institution, including a total number of 34 RCC patients and 44 recurrent RCC tumors in different locations (kidney, lung, adrenal gland, liver, pancreas, pararenal and other) using microwave ablation, radiofrequency ablation, cryoablation and laser ablation. The estimated time to local and distant tumor progression after treatment were 22.53 ± 5.61 months and 24.23 ± 4.47 months, respectively. Systemic treatment was initiated in 10/34 (29%) treated patients with a mean time-to-systemic-therapy of 40.92 ± 23.98 months. Primary technical success was achieved in all cases and patients while the primary efficacy rate was achieved in 43/44 (98%) cases and 33/34 (97%) patients, respectively, with a secondary technical success and efficacy rate of 100%. At a mean follow-up of 57.52 months ± 27.86 months, local tumor progression occurred in 3/44 (7%) cases and distant progression in 25/34 (74%) patients. No significant complications occurred. Image-guided ablations can play a role in helping to better control recurrent disease, avoiding or delaying the administration of systemic therapies and their significant adverse effects.

## 1. Introduction

Renal cell carcinoma (RCC) represents one of the most commonly diagnosed tumors in men and women, accounting for about 2–3% of all adult malignancies worldwide [1]. RCC incidence has risen in recent decades mainly because of the increased use of imaging techniques and it is expected to further increase, especially in developing countries, through 2030 [2]. According to the American Cancer Society estimates, the 5-year survival rate for localized forms of RCCs is 93%, which decreases to 71% when regional lymph nodes or surrounding tissues and organs are involved, finally reaching a dismal 14% when distant metastases occur [3]. Currently, in the management of RCC patients, an image-guided ablation therapy is increasingly offered as an alternative when surgery is not an option because of comorbidities, as well as advanced age, metastatic disease or surgery refusal. Along this line, a wide spectrum of local treatments, such as microwave ablation (MWA), radiofrequency ablation (RFA), cryoablation (CA), high intensity focused ultrasound (HIFU) and laser ablation, can play an important role throughout all stages of RCC treatment. Because of this, a multidisciplinary approach to recurrent RCC (rRCC) patients, including image-guided ablation treatments, has become increasingly required, aiming to provide a safe and effective treatment strategy. In particular, image-guided ablations can be useful in the multimodal approach to oligometastatic or oligoprogressive patients in order to ﻿avoid or delay the start of systemic treatments and their toxicity profiles. The aim of the present study is to report the results of the application of image-guided ablations in the treatment of patients with rRCCs, particularly focusing on the possibility of avoiding or delaying systemic treatments in those patients.

## 2. Materials and Methods

A retrospective analysis of electronic records and imaging databases was undertaken to identify patients who underwent the image-guided ablation of rRCCs. Institutional review board approval was obtained and patients’ informed consent was waived.

Indication to image-guided ablation was taken into a multidisciplinary meeting involving urologists, radiologists, oncologists and radiation therapists. The percutaneous approach was chosen taking into account age, comorbidities, tumor location, size and its proximity to critical non-target structures. The ablation technology adopted depended on various factors such as preference and familiarity of the first operator, morphological characteristics and location of the tumor, intraprocedural morphological results and technology availability. All procedures were performed by different interventional radiologists, at least one of these with more than 10 years of experience.

### 2.1. Procedures

All ablation procedures were performed in one of two dedicated angio suites. One room was equipped with a C-arm (Ziehm Vision RFD Hybrid Edition, Ziehm Vision, Nuremberg, Germany), a computed tomography (CT) (GE Lightspeed, GE Healthcare, Chicago, IL, USA) and an ultrasound (US) machine (GE E9, GE Healthcare, Chicago, IL, USA) equipped with a dedicated fusion imaging software. The other room was equipped with a hybrid angiography CT solution (ALPHENIX 4D CT, Canon Medical Systems Corporation, Kanagawa, Japan) and a US machine (GE E9, GE Healthcare, Chicago, IL, USA) equipped with dedicated fusion imaging software. All procedures were performed under general anesthesia. Careful patient positioning planning was performed and the most advantageous decubitus was secured with the help of dedicated devices such as a vacuum mattress to immobilize the patient body during the procedure. A contrast enhanced CT (CECT) scan was then performed to evaluate the tumor and allow for fusion imaging with US images. On the basis of the size and geometry of the tumor and its relationships with adjacent organs, the best access strategy was decided. Adjunct procedures to displace sensitive structures, such as hydrodissection, were applied according to interventional radiologist’s judgment. The ablative devices were than inserted under real time US/CT fusion imaging guidance, and correct device position was confirmed with a CT scan. Regarding RF, a LeVeen 17 G umbrella-shaped electrode (LeVeen CoAcces RFA, Boston Scientific, Marlborough, MA, USA, needle electrode) was adopted using increasing power up to roll-off twice. Regarding MW, a 13 G internally cooled antenna (Emprint, Medtronic, Minneapolis, MN, USA) was used. Power and time were established according to the manufacturer indications and to the size and shape of the tumor to be treated on a case-by-case basis. Regarding laser ablation, under CT-US fusion imaging, a 21 g needle was inserted at the level of the lesion to be treated and its correct position confirmed by an unenhanced CT. Then, a 0.3 mm laser fiber was introduced into the 21 g needle and thermal ablation was performed with a commercially available semi-conductor diode laser system with a wavelength of 1064 nm (Echolaser, Elesta Srl., Florence, Italy). A fixed 3 W power protocol was used with a total energy delivered of 1200–1800 J for a single illumination. Cryoablation was performed using the ProSense system (Ice-Cure Medical Ltd., Caesarea, Israel). Two to three cycles of freezing with intervening passive thaw were applied according to manufacturer indications and the lesions’ size. After ablation, independently from the adopted technique, a CECT was always performed to control for the immediate result and eventual presence of complications. In the case of a suspicious persistence of pathological tissue beyond the ablation area, device repositioning and a further ablation were performed in the same manner as previously described. For all treatments, a CECT within 24 h was performed to evaluate early treatment outcomes and complications. Finally, if no complications were observed, the patient was discharged the day after.

### 2.2. Follow-Up Protocol

Follow-ups were performed by CECT or contrast-enhanced MRI at established intervals (6 weeks, 3, 6, 12 months) following the first treatment and subsequently on an annual basis by the same group of interventional radiologists who routinely take care of these patients.

### 2.3. Variables and Data Analysis

Technique feasibility was defined as the possibility to perform the ablation. In the case of any factor (e.g., the impossibility to perform general anesthesia, or to achieve a safe distance from critical structures with hydrodissection), the procedure was considered unfeasible. Technical success was considered as the tumor being treated according to the pre-established protocol and covered completely by the ablation zone, while technique efficacy was defined as complete ablation at 6 months from treatment. Primary efficacy rate was defined as the percentage of complete ablation following the initial treatment, whereas secondary efficacy rate was considered as the percentage to complete ablation, including eventually repeated ablations. The time to first recurrence was defined as the time from primary surgery to first recurrence. Tumor progression was evaluated as local (recurrence at the site of ablation) or distant (development of new metastases), and the time to local and distant tumor progression (local-TTP, distant-TTP) were estimated with the Kaplan–Meier method. Time to systemic therapy initiation was evaluated, accounting for the time from the first ablation to the time of first systemic therapy prescription. The overall survival (OS) and disease-free survival (DFS) were estimated with the Kaplan–Meier method. Complications were recorded and classified in minor, major and severe adverse events (AEs) according to the Society of Interventional Radiology classification [4,5]. Statistical analyses were performed using commercial software package XLSTAT (Addinsoft, New York, NY, USA). Results were reported as means, standard deviation (SD), medians and ranges for the quantitative variables and percentages for the categorical variables.

## 3. Results

Between January 2012 and October 2021, a total number of 34 patients (mean patients age 64.5 ± 10.4, 10 F, 24 M) underwent the treatment of 44 metachronous tumors (mean number of tumors per patient: 1.29 ± 0.40; median: 1; range 1–2) in 35 treatments. A histological diagnosis of both the primary renal tumors and recurrent tumors were available for all patients that underwent image-guided ablation. All patients were oligometastatic, with 24/34 (71%) accounting for one lesion site and 10/34 (29%) for two lesion sites. The 44 treated lesions included mostly the contralateral kidney (*n* = 23/44; 52%), lung (*n* = 5/44; 11%), adrenal glands (*n* = 4/44; 9%), liver (*n* = 3/44; 7%), pancreas (*n* = 1/44; 2%) and five soft-tissue sites: pararenal space (*n* = 3/44; 7%), perisplenic space (*n* = 2/44; 5%), retroperitoneal space (*n* = 1/44; 2%), abdominal wall (*n* = 1/44; 2%) and subdiaphragmatic space (*n* = 1/44; 2%). Regarding the histologic subtypes of treated patients, the absolute majority of them were RCCs (33/34; 97%) with 32/33 (97%) accounting for clear cell RCCs and 1/33 (3%) patients for type 1 papillary tumors.

Tumor grade was determined using the Fuhrman grading system with the majority of treated lesions (31/44; 71%) accounting for Grade 2 and 3. The clinicopathological characteristics of treated patients are summarized in Table 1.

At a mean follow-up time of 57.52 ± 27.86 months (median 59.80; range, 8.67–99.47 months), local tumor progression (LTP) was identified in 3/44 (7%) lesions that were successfully retreated with percutaneous ablation.

**Figure 1:**
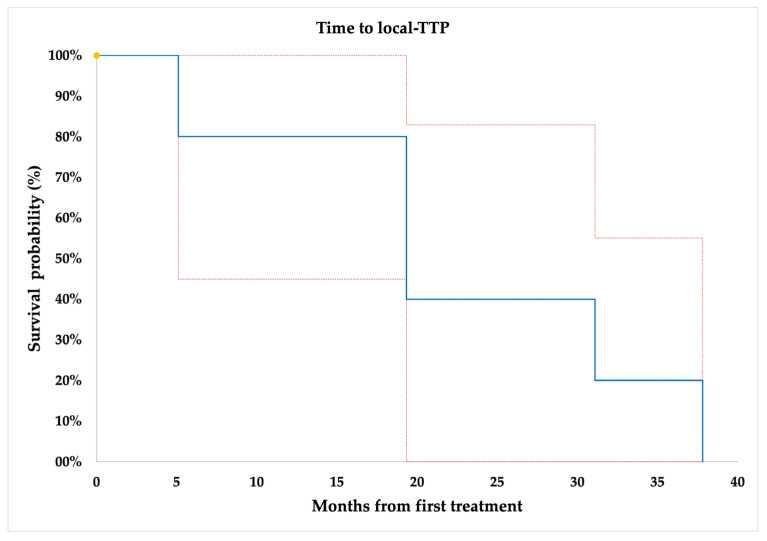
Time to local tumor progression (TTP), per-lesion analysis (*n* = 44). Yellow dots = censored data.

The estimated mean time to local-TTP was 22.53 ± 5.61 months (Figure 1).

**Figure 2:**
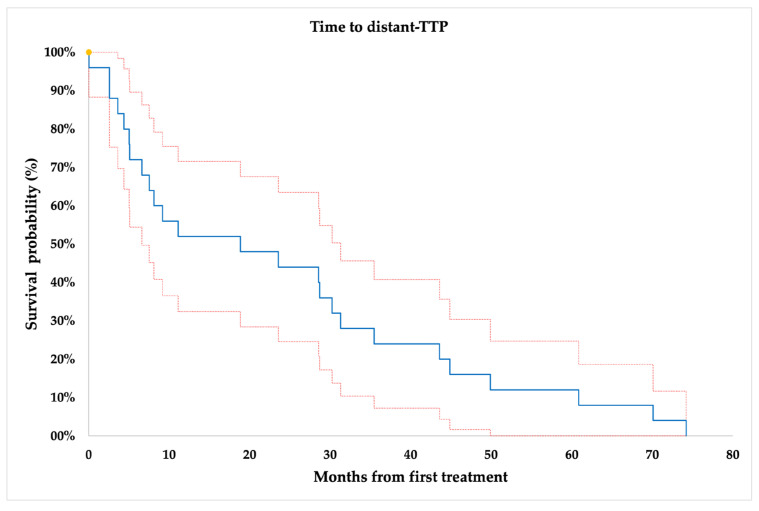
Time to distant tumor progression (TTP), per-patient analysis (*n* = 34). Yellow dots = censored data.

Regarding patients’ overall progression, 25/34 (74%) of the successfully treated patients showed further disease progression with a mean time to distant-TTP of 24.23 ± 4.47 months (Figure 2).

Systemic treatment was initiated in 10/34 (29%) treated patients, with a mean time-to-systemic-therapy of 40.92 ± 23.98 months.

**Figure 3:**
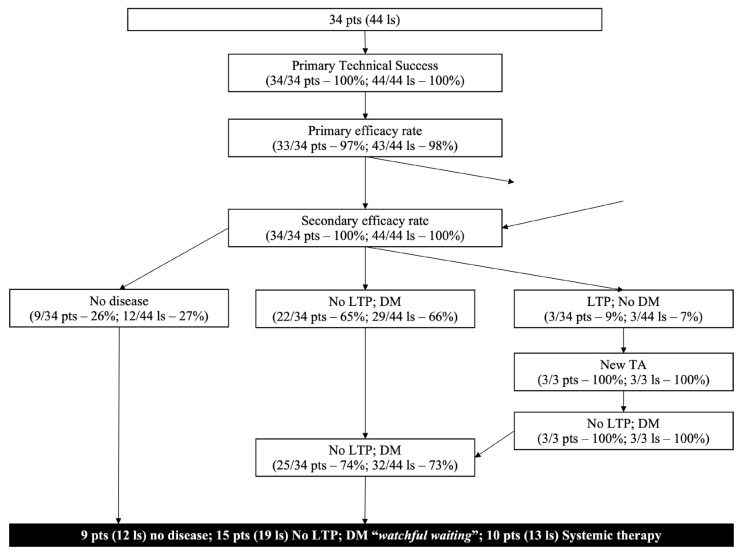
Flow chart of the course of 34 patients and 44 lesions from rRCC reporting treatment to last follow-up. Pts patient; Ls lesions; LTP local tumor progression, DM distant metastasis, TA tumor ablation.

A flow chart of the course of 34 patients and 44 rRCC lesions is available in Figure 3.

**Figure 4:**
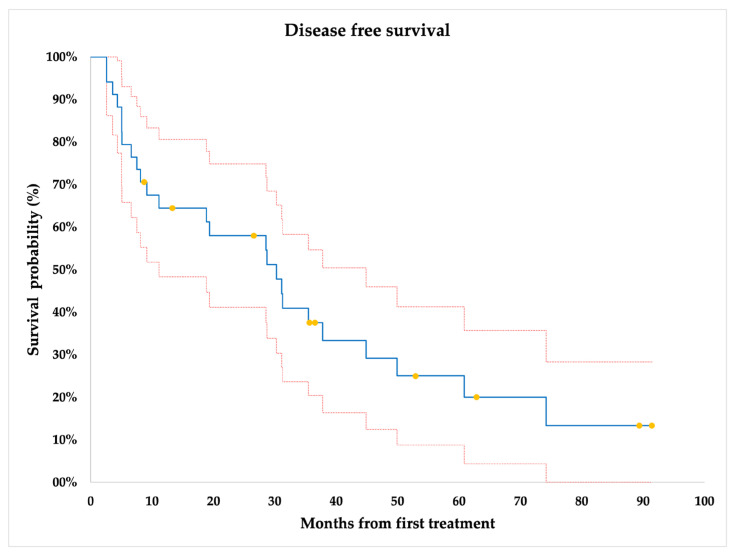
Disease-free survival (DFS) of the whole cohort. Yellow dots = censored data.

As reported in Figure 4, the mean DFS was 33.08 ± 4.75 months.

**Figure 5:**
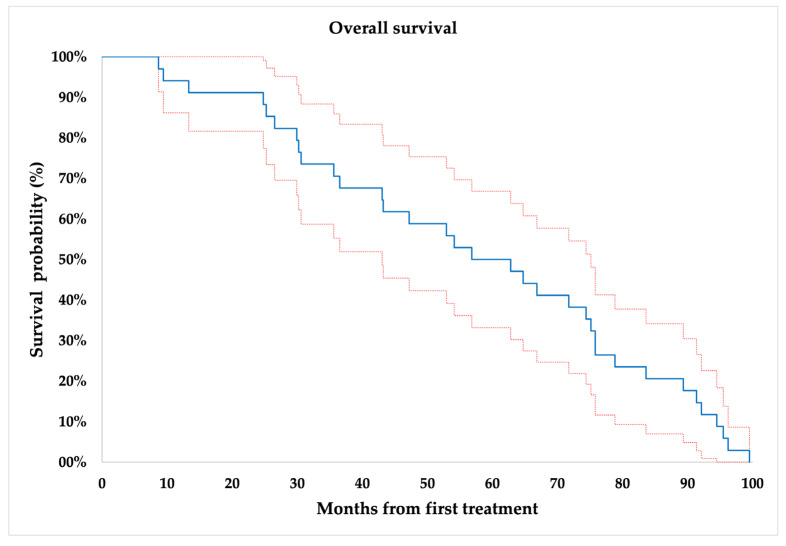
Overall survival of the whole cohort.

As shown in Figure 5, the mean OS was 57.52 ± 4.77 months.

Primary technical success was achieved in all lesions and patients while primary efficacy rate was achieved in 43/44 (98%) lesions and 33/34 (97%) patients due to a residual unablated tumor at first imaging follow-up. In this case, a second treatment was successfully performed and, therefore, secondary technical success and efficacy rate were achieved in all initially included lesions (44/44; 100%) and patients (34/34; 100%).

A total amount of nine complications occurred over 44 treated lesions (20%) in 5/34 (15%) patients. No minor complications (A–B) occurred. Major complications (C–F) accounted for 9/9 (100%) treated lesions. No severe treatment-related AEs (E–F) occurred. Complications are listed in Table 2.

**Figure 6:**
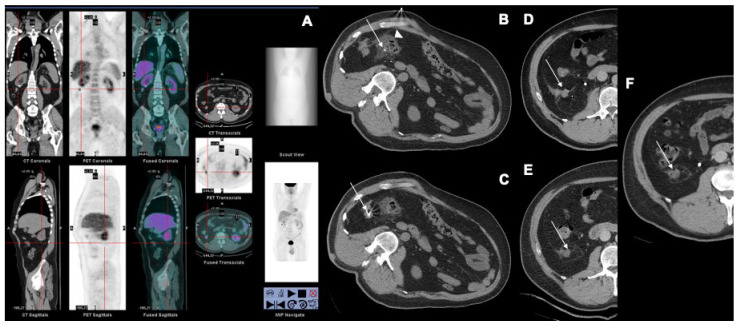
A 52-year-old patient with a right-sided retroperitoneal recurrence 2 years after a right-kidney partial nephrectomy, treated with MWA. (**A**) A PET-CT scan performed a few days before treatment showing a right retroperitoneal nodule with vivid FDG uptake. (**B**) Non-enhanced CT performed during the procedure showing hydrodissection performed with saline solution to create a safety margin (arrowhead) between the tumor (arrow) and the adjacent colon. (**C**) Non-enhanced CT performed during the procedure illustrating the microwave antenna at the lesion level (arrow). (**D**–**F**) Contrast-enhanced CT performed at 24 h, 1.5 months and 1 year showing complete ablation of the nodule (arrow) without contrast enhancement and no complications.

Images from a patient treated with MWA for a retroperitoneal recurrence are shown in Figure 6.

## 4. Discussion

Approximately 20–30% of RCC patients have metastatic disease at diagnosis (synchronus disease), while about 20–40% of cases can develop distant metastases after the initial diagnosis (metachronous disease) [6]. Of interest, 57 to 65% of RCC patients exhibit single sites of metastatic lesions, being the number of metastatic sites inversely related to the patients’ age [7]. Recent estimates suggest that around 25% of RCC patients with metachronous metastatic disease could be candidates for local treatments [8], and the most common metastatic sites to which we have to pay attention deal with the lung (45.2%), bone (29.5%), lymph nodes (21.8%), liver (20.3%), adrenal (8.9%) and brain districts (8.1%) [9,10].

In the last decade, many novel therapeutic options combining multikinase inhibitors and immune checkpoint inhibitors [11] have been investigated in metastatic RCC (mRCC) patients. Unfortunately, the variability of trial designs and different drug combinations have made difficult to establish a unique first-line treatment [12]. Within this scenario, conservative or radical surgery still persist as the standard of care for the treatment of patients with localized or locally advanced RCCs [13,14]. Particularly, in isolated retroperitoneal recurrences after nephrectomy, a careful surgical resection is still supported [15], but when a further oligometastatic progression appears, image-guided ablations can play a role, deferring the need for a systemic treatment [16,17,18]. The current literature suggests that a locoregional recurrence after radical primary surgery most often behaves as a systemic disease, with a lot of patients presenting distant metastases, while only a few of them have exclusive locoregional recurrence. The latter is the ideal condition for image-guided ablations [18,19].

Image-guided ablations, such as MWA, RFA, MWA plus RFA, CA and laser ablation have significantly progressed over the past years and they represent a useful toolbox for a multimodal approach to this disease [15,20,21,22,23,24]. The most common shared indications concern localized or small RCCs, unable to undertake surgery due to comorbidities, bilateral tumors or locoregional recurrences.

Our data present a unique perspective of exclusive image-guided ablations applied to the treatment of rRCC patients without including other local treatments such as stereotactic radiosurgery (SRS), conventional radiotherapy (CRT) or stereotactic body radiotherapy (SBRT). Our series included an heterogenous group of oligo-metastatic metachronous rRCC patients with different tumor locations encompassing the contralateral kidney, lung, adrenal gland, liver, pancreas and pararenal lesions.

Regarding the adoption of different ablation techniques, both RFA and CA are advised by current guidelines for the treatment of RCC and are considered equivalent in terms of excellent technical success rates and low tumor recurrence rates, even in cases of repeated treatments [25,26,27]. Moreover, no significant outcome differences between the two techniques regarding complications, metastasis-free rates and cancer-specific survival have been reported [25,26,28]. Regarding MWA, due to its more recent development compared to RFA and CA, limited studies, lacking in mid- and long-term follow-ups, are currently available. However, MWA presents practical advantages compared to RFA, with shorter procedural times and a reduced heat-sink effect, and also providing larger ablation zones [29,30,31]. In our institution, due to a larger experience with MWA in different organs and locations, we usually adopt this technique when technically feasible also for RCC lesions, without observing a higher recurrence rate vs. other ablation techniques [22,32,33,34].

These technologies have been subsequently extended to the treatment of recurrent and metastatic lesions even if the current literature is still limited. A study from Bang et al. comprising a group of 27 patients (67% with intermediate risk) treated with cryoablation for 72 tumors in heterogeneous locations, reported a median OS of 32.3 months vs. 57.52 months in our series. Also, even if the recurrence rate was reduced (1% vs. 7%), 22% of patients received a systemic therapy before the ablation treatment [35]. Another paper by Welch et al., involving 82 RCC heterogeneous lesions in 61 patients treated with different image-guided ablation techniques, reported a technical failure in 4.9% of treatments with a comparable recurrence rate (7.9% vs. 7% in our series) and a good 3-year OS of 76%. However, also in this case, 26% and 4% of patients received systemic therapy or radiotherapy before ablation, respectively [36].

In our series, the treatments were feasible in all cases and locations with high technical success and efficacy rates, supporting the role of image-guided ablations as flexible, safe and effective treatment options for these patients. Moreover, at the same time no perioperative systemic therapy was added as in other papers dealing with surgical treatments of RCC local recurrences [37].

The quite-long median time to recurrence from the initial surgery in our series (36 months; range, 0–167 months) gives a rationale to maintain such patients under follow-up after the first initial nephrectomy, in order to identify those who could benefit from early image-guided ablations [37,38,39]. Therefore, we also applied a strict follow-up after image-guided ablation, identifying at a mean imaging follow-up time of 57.52 ± 27.86 months (median 59.80; range, 8.67–99.47 months) an LTP in 3/44 (7%) of successfully treated tumor lesions. Then, the estimated mean local-TTP was 22.53 ± 5.61 months, which is comparable to recently published papers that included surgical resection as local treatment as well [9,18,40]. Our results are in line with a surgical series from Ray et al. of 30 patients who underwent resection for 26 local and regional RCC recurrences, showing a secondary recurrence in 53.8% of patients with a median time to recurrence of 11.5 months (range 7–29) [18]. Moreover, in a recent systemic review regarding surgical metastasectomy of metastatic RCCs in various organs, a significant longer median OS or cancer-specific survival (median 40.8 months) was reported after complete metastasectomy compared with incomplete or no metastasectomy, highlighting the role of local treatments in terms of survival outcome improvement and the delaying of systemic treatment with its associated toxicity [9]. As described in the surgical literature, estimates reports that 25% of patients with metachronous metastasis might be candidates for local treatment [8]; however, patient selection can be complex because of the heterogeneous course of RCC, surgical resectability and anatomical access. According to this, establishing a role for other types of local therapies for rRCCs, especially image-guided ablations, is of timely importance [41,42]. After image-guided ablation, our data showed that progression at distance occurred in 25/34 (74%) patients at a mean estimated time of 24.23 ± 4.47 months. Systemic treatment was started in 10/34 (29%) of our patients, at a mean time of 40.92 ± 23.98 months after the ablation. Therefore, the start of a systemic therapy could be avoided in the large majority of cases, and when required it could be postponed for more than three years, indicating an indolent course of a patients’ disease after image-guided ablations. Moreover, systemic therapies, mainly consisting of kinase inhibitors such as cabozantinib or sunitinib, and more recently immunotherapy such as nivolumab or pembrolizumab, are burdened by significant adverse effects, most frequently of advanced grade, with a possible long-term impact on daily patients’ quality of life [43,44,45,46,47]. It is worth mentioning that the complication rate involved 20% of all treated lesions, a finding that is lower than comparable locoregional surgical treatments and systemic therapies [9,48].

In our series of rRCC patients, the mean OS was 57.52 ± 4.77 months, which was comparable to different data reported in the review of Dabestani et al., in which many studies on treatments of advanced RCC patients included metastasectomy and radiotherapy modalities [9].

Overall, we believe that image-guided ablation can play a role as a less invasive treatment in rRCCs, especially in metachronous oligo-metastatic patients with a low-to-intermediate Fuhrman grade and long time to the first recurrence [49], accounting for low complication rates, low morbidity risk with repeated procedures [50] and a prominent role in delaying systemic treatments with their associated toxicity.

Finally, our study has some limitations, including the retrospective study design, the small sample size and its heterogeneity with different metastatic locations and multiple ablation methods adopted, which could not allow a per-location and per-treatment analysis. Further studies with larger groups and longer follow ups are needed in the future to establish the ideal location site and ablation technique for these recurrences. Notwithstanding, to date, no prospective trials regarding image-guided ablations for rRCC patients have been performed and, to the best of our knowledge, this is currently one of the largest series of rRCC treated with image-guided ablation. From this, our results can be considered useful for generating hypotheses and planning further studies to shed light on the role of image-guided ablations in the treatment of rRCC patients.

## 5. Conclusions

Over the last years, the therapeutic options for the treatment of mRCC patients have significantly changed, especially with the introduction of immune-combination strategies that became the standard of care in this disease setting, extending patients’ survival at expenses of significant adverse effects.

Therefore, establishing a role for image-guided ablations that can help to better control the disease and avoid or delay the administration of systemic therapies in carefully selected rRCC patients is of paramount importance.

## Figures and Tables

**Table 1:** Characteristics of 34 patients who underwent image-guided ablation of rRCC (*n* = 44) in 35 treatment sessions.

Characteristic	Value
**Mean Age (y)**	64.5 ± SD 10.4
**Sex**	
- M	24/34 (71%)
- F	10/34 (29%)
**Treated tumors’ location**	
- Kidney	23/44 (52%)
- Lung	5/44 (11%)
- Adrenal gland	4/44 (9%)
- Liver	3/44 (7%)
- Pancreas	1/44 (2%)
- Pararenal	3/44 (7%)
- Perisplenic	2/44 (5%)
- Retroperitoneal	1/44 (2%)
- Abdominal wall	1/44 (2%)
- Subdiaphragmatic	1/44 (2%)
***Per-patient’s* lesions**	
1 lesion site	24/34 (71%)
2 lesion sites	10/34 (29%)
**Histologic subtype**	
- RCC	33/34 (97%)
- Clear cells	32/33 (97%)
- Papillary (type 1)	1/33 (3%)
- Not-defined RCC	1/34 (3%)
**Fuhrman grading scheme**	
- Grade 1	1/44 (2%)
- Grade 2	21/44 (48%)
- Grade 3	10/44 (23%)
- Grade 4	2/44 (5%)
- Not available	10/44 (23%)
**Adopted ablation techniques**	
MWA	26/35 (74%)
RFA	5/35 (14%)
MWA + RFA	1/35 (3%)
Laser	2/35 (6%)
Cryoablation	1/35 (3%)

Image-guided ablations were feasible in all cases. The median time to first recurrence following treatment for primary RCC was 36 months (range, 0–167 months).

**Table 2:** Complications among patients (*n* = 34) who underwent image-guided ablation of rRCC (*n* = 44), classified according to SIR standards.

Grading	Number (%)		Type of Complication	Details
**A**	No minor complications			
**B**				
**D**	9/44(20%)	1/9 (11%)	Pancreatic fistula	7-day hospitalization
		8/9 (88%)	Perilesional hematoma	3–5 days hospitalization

## Data Availability

The data that support the findings of this study are available on request from the corresponding author.
